# Developing a Mobile App for Monitoring Medical Record Changes Using Blockchain: Development and Usability Study

**DOI:** 10.2196/19657

**Published:** 2020-08-14

**Authors:** MinDong Sung, SungJun Park, Sungjae Jung, Eunsol Lee, Jaehoon Lee, Yu Rang Park

**Affiliations:** 1 Department of Biomedical Systems Informatics Yonsei University College of Medicine Seoul Republic of Korea; 2 Medibloc Inc Seoul Republic of Korea; 3 Intermountain Healthcare Salt Lake City, UT United States; 4 Department of Biomedical informatics University of Utah Salt Lake City, UT United States

**Keywords:** blockchain, monitoring app, clinical documents

## Abstract

**Background:**

Although we are living in an era of transparency, medical documents are often still difficult to access. Blockchain technology allows records to be both immutable and transparent.

**Objective:**

Using blockchain technology, the aim of this study was to develop a medical document monitoring system that informs patients of changes to their medical documents. We then examined whether patients can effectively verify the monitoring of their primary care clinical medical records in a system based on blockchain technology.

**Methods:**

We enrolled participants who visited two primary care clinics in Korea. Three substudies were performed: (1) a survey of the recognition of blockchain medical records changes and the digital literacy of participants; (2) an observational study on participants using the blockchain-based mobile alert app; and (3) a usability survey study. The participants’ medical documents were profiled with HL7 Fast Healthcare Interoperability Resources, hashed, and transacted to the blockchain. The app checked the changes in the documents by querying the blockchain.

**Results:**

A total of 70 participants were enrolled in this study. Considering their recognition of changes to their medical records, participants tended to not allow these changes. Participants also generally expressed a desire for a medical record monitoring system. Concerning digital literacy, most questions were answered with “good,” indicating fair digital literacy. In the second survey, only 44 participants—those who logged into the app more than once and used the app for more than 28 days—were included in the analysis to determine whether they exhibited usage patterns. The app was accessed a mean of 5.1 (SD 2.6) times for 33.6 (SD 10.0) days. The mean System Usability Scale score was 63.21 (SD 25.06), which indicated satisfactory usability.

**Conclusions:**

Patients showed great interest in a blockchain-based system to monitor changes in their medical records. The blockchain system is useful for informing patients of changes in their records via the app without uploading the medical record itself to the network. This ensures the transparency of medical records as well as patient empowerment.

## Introduction

We currently live in an era of data management and often pursue goals of open access and transparency, which means that anyone can usually find information promptly based on their specific needs. One exception to such transparency is medical data [1]; although medical records entered by clinicians and stored in clinical information systems legally belong to patients [2,3], many patients realistically find it difficult to gain full and transparent access to their own medical records.

Patient empowerment has long been emphasized and was recently highlighted in the “2020-2025 Federal Health IT Strategic Plan” from the Office of the National Coordinator for Health Information Technology (ONC). To improve patient empowerment, electronic health records (EHRs) should be shared with patients. However, there are several potential threats to EHRs that could undermine trust in data on these systems. First, records can be altered or lost, either accidentally or intentionally, such as through hacking. Even though redundancy exists in database systems, these redundancies are often obscure to outside observers. Second, data can be fabricated or manipulated by medical staff intent on committing fraud. A possible solution to overcoming these dilemmas is blockchain technology, which uses distributed and cryptographically secure ledgers to ensure immutability, transparency, and decentralization. Bitcoin is a well-known example of blockchain in the field of cryptocurrency [4]. Blockchain also provides logs of when data are created, changed, or deleted. Thus, providing all data logs can overcome the two primary threats to EHRs.

Some previous studies have reported the implementation of blockchain to health care [5]. Most of these approaches focus on storing and sharing institutional medical data between EHRs [6-9] and personal health records (PHRs) [10-12]. In addition, blockchain has been implemented for sharing and storing clinical trial data [13,14]. Most of these existing works proposed a well-organized architecture or frameworks and a few demonstrated the performance of the prototype developed during the study. However, no study has yet revealed the actual benefits of developing an EHR system using blockchain. We could consider that such a system using blockchain, which features characteristics of transparency and immutability, would be transparent and immutable.

Therefore, in this study, we used blockchain technology to develop a medical document monitoring system that notifies patients of changes in their medical records. The system was then tested with simulation data and the proof-of-concept study was performed in primary care clinics in Korea.

## Methods

### Study Design

This is a proof-of-concept study consisting of three substudies: (1) a survey of the recognition of blockchain medical records changes and the digital literacy of participants; (2) an observational study on participants using the blockchain-based mobile alert app; and (3) a usability survey study. The study was approved by the Institutional Review Board of Yonsei University Health System (Y-2019-0127) and all participants provided informed consent.

Before proceeding with the design and development of the mobile app, since the EHR systems used in each hospital setting differ, the documents used in the EHR systems were profiled using HL7 Fast Healthcare Interoperability Resources (FHIR; see Multimedia Appendix 1) [15,16]. Although it is reasonable that patients are notified of any changes in medical records, patients might be overwhelmed by too many notifications whenever any change occurs. Moreover, to improve the usability of the mobile app, changes should be summarized. Therefore, medical documents were divided into three types according to their importance and impact, and the mobile app was designed so that patients are notified of only high-risk changes in documents according to the following three risk levels: risk 1, medical information and other critical items that should not be changed; risk 2, medical information and other items that are allowed to be changed; and risk 3, nonmedical information. These risk levels were also considered in the profiles created.

**Figure 1:**
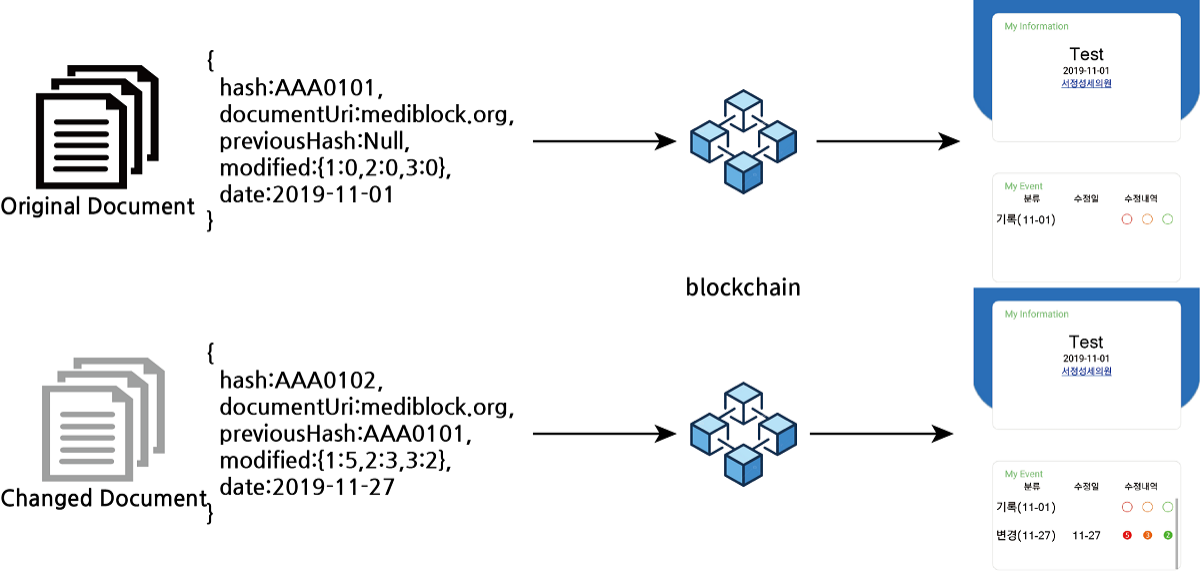
Structure of the medical document monitoring system.

The mobile app used in this study leverages the blockchain network MediBloc Panacea [17] that was developed based on the Tendermint blockchain [18]. The blockchain uses the delegated proof-of-stake method implemented by the practical Byzantine fault tolerance algorithm to create blocks. In this system, “delegated” refers to delegated nodes that perform and validate transactions and blocks. Validators are selected through voting. Normally, one block is generated per second, and all transaction history transmitted over the network is stored in the generated block. Further, similar to other blockchains, once created, blocks cannot be reversed. We used four different properties in the blockchain transaction: (1) writer of the transaction, which can only be specific clinics; (2) topic that is assigned per individual patient; (3) key; and (4) value. Key and value have the following five attributes: (1) hash value for medical documents, (2) document URL for the FHIR profile in which the document was transformed, (3) hash value before the medical document was changed, (4) risk and number of documents with changes, and (5) date to represent when the document was created. As soon as the document is created, the health information system spontaneously hashes and transacts the metadata of the document to the blockchain network (Figure 1). The mobile app provides users with logs of changes in medical records. The app has been available on the Google Play Store [19] since October 23, 2019.

Before the proof-of-concept study was deployed, we simulated the app to evaluate how it captures fake medical records caused by fraudulent actions. We created five fake medical documents for the purpose of simulation. All except for one dataset were assumed to have changes in the documents. The risk levels of the changes were set differently for each dataset. We transacted the datasets and checked how the mobile app worked.

The study was conducted at two primary care clinics specializing in pediatrics in Korea. Only outpatients of these two clinics were enrolled in the study. Anyone who visited the selected clinics was eligible to participate in the study. Because young patients were not interested in their own medical records, whereas their guardians were more interested, the guardians of patients enrolled in the study on their behalf.

When patients launch the mobile app from their devices, the user is authenticated according to a username and password. After login, the patient can see their own profile and events, which include when the records were created as well as which and how many items at high risk were changed. This information was obtained by querying blockchain using Owner and Topic values that were stored in the backend server of the mobile app.

### Substudies

**Figure 2:**
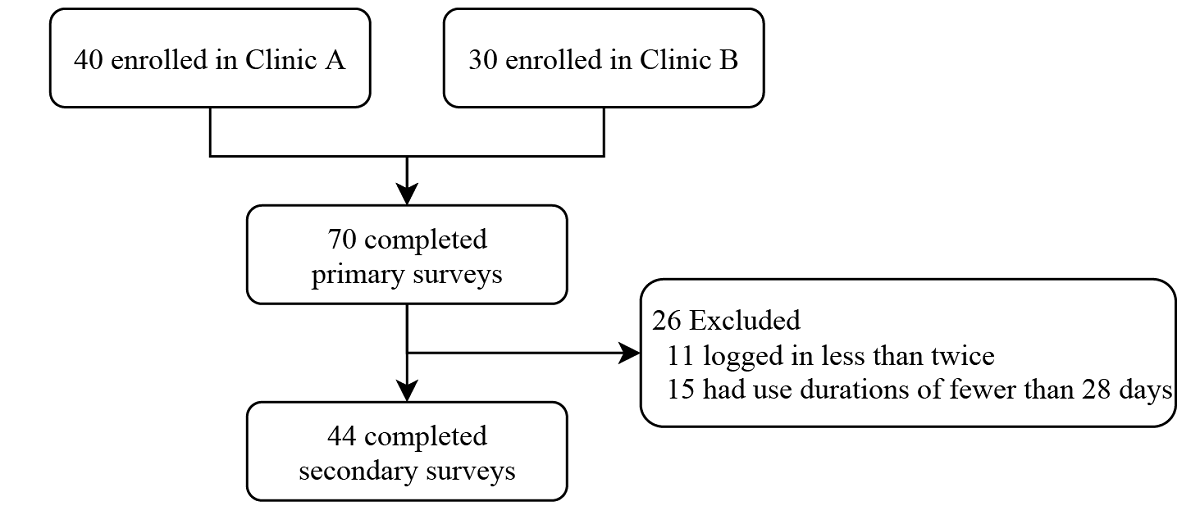
Flow diagram of study participants.

To investigate participants’ level of recognition of blockchain, medical records changes, and their digital literacy, questionnaires were completed by all participants (Figure 2). The primary survey consisted of items regarding the recognition of blockchain, thoughts and experiences of changes in medical records, and digital literacy. There were six questions that measured blockchain recognition; these were based on virtual currency questions from a 2014 Survey of Consumer Payment Choice [20]. The second part of the questionnaires consisted of four questions. The first two questions were related to the recognition of medical record changes and the need for a monitoring system for medical record changes. These two questions were answered on a 5-point response scale. The other two questions addressed the participants’ experience of medical record changes. The third part, digital literacy, consisted of 10 questions answered on a 5-point response scale. These questions were taken from other digital literacy questions [21], modified for the mobile app, and condensed to 10 questions. A higher score represents a higher level of digital health literacy, except in the case of two questions. The questionnaire ended with demographic characteristics (age, sex, and occupation). All 5-point questions were answered, with responses ranging from very negative to very positive (ranging between 1 and 5).

Usage patterns of the app were measured by the number of logins, duration that indicated the difference in days between the first and the last logins, and an event log, which indicated how many items were changed and how important they were. These usage patterns were observed in participants who logged in more than once and for a duration of more than 28 days.

After 28 days of using the app, participants were invited via the app to take part in the secondary survey, which included a System Usability Scale (SUS) survey. For the evaluation of learnability and usability, we used the modified SUS, a reliable, low-cost usability scale that can be used for global assessments of systems usability (see Multimedia Appendix 2) [22,23]. Bangor et al [23] described the adjective ratings associated with SUS scores as follows: worst possible (mean SUS score 25), poor (39.17), satisfactory (52.01), good (72.75), excellent (85.58), and best possible (100).

### Statistical Analysis

Categorical variables are presented as numbers and percentages and were compared using the Chi-square test and the Fisher exact test. Continuous variables are expressed as mean (SD) and were compared using the Student unpaired *t*-test, analysis of variance, Wilcoxon signed-rank test, or Mann-Whitney *U* test as appropriate. Surveys answered by a Likert-type scale and scores are expressed as the mode as well as numbers and percentages. All statistical analyses were performed using R version 3.6.1 (R Foundation for Statistical Computing, Vienna, Austria) and 2-tailed tests. Results with *P*<.05 were considered to be statistically significant.

## Results

Before the proof-of-concept study was performed, metadata from the 5 original documents and the 4 changed documents were transacted into the blockchain. The simulation metadata and transaction metadata are described in Multimedia Appendix 3. App screenshots are provided in Multimedia Appendix 4.

A total of 70 patients were enrolled in this study. Patient characteristics are shown in Table 1. There were more female patients, and the majority of patients were in their thirties. Thirty-two participants were guardians of the patients, and the occupation of nearly half of the participants was housework or parenting (Table 1).

**Table 1:** Demographic characteristics of participants.

Characteristic		Hospital A (n=40)		Hospital B (n=30)		Total (N=70)		*P* value	
**Sex, n (%)**								.29	
	Male		9 (23)		3 (10)		12 (17)		
	Female		31 (77)		27 (90)		58 (83)		
**Respondent, n (%)**								.47	
	Guardian		19 (47)		18 (60)		37 (53)		
	Patient		21 (53)		12 (40)		33 (47)		
**Age groups (years), n (%)**								.40	
	19-20		0 (0)		1 (3)		1 (1)		
	20-29		5 (12)		1 (3)		6 (9)		
	30-39		22 (55)		17 (57)		39 (56)		
	40-49		12 (30)		10 (33)		22 (31)		
	50-59		1 (3)		0 (0)		1 (1)		
	>60		0 (0)		1 (3)		1 (1)		
**Occupation, n (%)**								.24	
	Information technology		1 (3)		0 (0)		1 (1)		
	Office job		5 (12)		5 (17)		10 (14)		
	Management		2 (5)		0 (0)		2 (3)		
	Professional		4 (10)		3 (10)		7 (10)		
	Housework/parenting		13 (32)		17 (57)		30 (43)		
	Sales		2 (5)		0 (0)		2 (3)		
	Student		0 (0)		1 (3)		1 (1)		
	Unemployed		2 (5)		1 (3)		3 (4)		
	Other		11 (28)		3 (10)		14 (20)		

Recognition of blockchain concepts differed depending on the type of question (ie, whether it refers to bitcoin or blockchain). The majority of participants stated that they were aware of bitcoin, whereas less than half were aware of blockchain. Similarly, respondents were more familiar with bitcoin than with blockchain. However, respondents had less trust in bitcoin than in blockchain. In terms of the recognition of medical record changes, participants tended to not allow changes in their medical records. Subsequently, participants stated a need for a medical record monitoring system. There was only one participant who reported having experienced medical document changes in the first questionnaire. The medical records were changed to correct the wrong information entered previously. In terms of digital literacy, most of the questions were answered as “good” digital literacy (Table 2).

**Table 2:** Distribution, n (%), of primary survey responses and modes of Likert scale scores (N=70).

Survey question		1		2		3		4		5		Mode	
**Awareness of blockchain**													
	Have you heard of bitcoin?		63 (90)		7 (10)		0 (0)		0 (0)		0 (0)		1
	How familiar are you with bitcoin and how it works?		17 (24)		21 (30)		24 (34)		6 (8.6)		2 (2.9)		3
	How much do you trust bitcoin?		10 (14)		29 (41)		26 (37)		3 (4.3)		2 (2.9)		2
	Have you heard of blockchain?		32 (46)		38 (54)		0 (0)		0 (0)		0 (0)		2
	How familiar are you with blockchain and how it works?		37 (53)		14 (20)		10 (14)		7 (10.0)		2 (2.9)		1
	How much do you trust blockchain?		19 (27)		11 (16)		29 (41)		7 (10.0)		4 (5.7)		3
**Recognition of medical document changes**													
	Do you think changing medical records should be allowed?		42 (60)		7 (10)		11 (16)		9 (13)		1 (1)		1
	Do you think we need a medical records falsification monitoring system?		1 (1)		2 (3)		6 (9)		18 (26)		43 (61)		5
**Digital literacy^a^**													
	Can you use the internet?		1 (1)		5 (7)		18 (26)		29 (41)		17 (24)		4
	Can you use digital technology?		2 (3)		8 (11)		29 (41)		20 (29)		11 (16)		3
	Can you use the app well on your phone?		1 (1)		3 (4)		21 (30)		22 (31)		23 (33)		5
	Can you use the camera well on your phone?		1 (1)		1 (1)		6 (9)		15 (21)		47 (67)		5
	Can you download and install apps from your phone?		1 (1)		1 (1)		9 (13)		16 (23)		43 (61)		5
	I feel comfortable using digital technology		0 (0)		3 (4)		10 (14)		30 (43)		27 (39)		4
	I am active in learning digital technology		3 (4)		13 (19)		21 (30)		24 (34)		9 (13)		4
	I feel threatened when others talk about digital technology^b^		18 (26)		30 (43)		16 (23)		4 (6)		2 (3)		2
	I feel behind other people my age in terms of digital technology^b^		6 (9)		23 (33)		32 (46)		7 (10)		2 (3)		3
	I believe that it is important for me to learn how to use digital technology		0 (0)		3 (4)		17 (24)		20 (29)		30 (43)		5

^a^A high score represents a high level of digital literacy, unless otherwise indicated.

^b^A low score represents a high level of digital health literacy.

Only 44 participants were included in the analysis of usage patterns and the secondary survey. During the study period, the app had been accessed a mean of approximately 5 times. The duration of app use, which was indicated by the difference between the first login and the last login, was approximately 34 days. However, there were no medical document changes during this period. The mean SUS score indicated “satisfactory” usability. The number of logins and the duration were significantly higher in hospital A than in hospital B. Moreover, the duration was significantly different according to occupations. There were no document changes during the study period (Table 3).

**Table 3:** Usage patterns and System Usability Scale (SUS) scores of app users.

Group		Participants, n (%)	Number of logins, mean (SD)	Duration (days), mean (SD)	SUS score, mean (SD)
Total		44 (100)	5.10 (2.60)	33.60 (10.00)	64.60 (16.00)
**Hospital**					
	Hospital A	24 (55)	5.88 (2.71)	34.63 (11.14)	67.50 (16.32)
	Hospital B	20 (45)	4.10 (2.07)	32.34 (8.55)	61.12 (15.36)
	*P* value^a^		.02	.05	.16
**Gender**					
	Male	7 (16)	4.57 (2.94)	31.12 (3.09)	68.21 (19.08)
	Female	37 (84)	5.16 (2.53)	34.06 (10.8)	63.92 (15.6)
	*P* value		.45	.98	.54
**Age group (years)**					
	20-30	6 (14)	6.50 (2.88)	36.95 (13.98)	67.08 (15.61)
	30-40	23 (52)	4.87 (2.85)	34.52 (11.21)	63.48 (17.4)
	40-50	14 (32)	4.86 (1.96)	30.86 (5.2)	65.54 (15.42)
	50-60^b^	1 (2)	4.00	30.09	62.50
	*P* value		.49	.38	.90
**Respondent**					
	Guardian	27 (61)	4.70 (2.61)	35.29 (12.33)	67.13 (17.29)
	Patient	17 (39)	5.65 (2.47)	30.89 (3.03)	60.59 (13.3)
	*P* value		.15	.57	.19^c^
**Occupation**					
	Housework/parenting	23(52)	4.96 (2.69)	31.44 (7.84)	63.15 (16.52)
	Professional	4 (9)	4.25 (2.22)	29.44 (1.17)	54.38 (2.39)
	Sales	2 (5)	3.00 (1.41)	33.46 (5.01)	57.50 (21.21)
	Office job	7 (16)	5.57 (2.51)	41.30 (14.53)	74.29 (19.02)
	Unemployed	2 (5)	6.00 (4.24)	30.04 (0.10)	72.50 (3.54)
	Other	6 (14)	5.83 (2.64)	36.82 (13.95)	65.42 (14.44)
	*P* value		.70	.03	.40^d^

^a^Mann-Whitney *U* test and Kruskal-Wallis rank-sum test were used to calculate *P* values between two groups and three groups, respectively, unless otherwise indicated.

^b^SD not available since there is only one value.

^c^Calculated using the *t* test.

^d^Calculated using analysis of variance.

## Discussion

### Principal Findings

This proof-of-concept study applied a blockchain-based medical document monitoring system in two primary care clinics. Although there was a lack of recognition of the concept of blockchain (compared with bitcoin), participants’ trust level in blockchain was higher than that in bitcoin. Moreover, although there were few people who had experienced changes in their medical documents, the participants considered that medical records should not be changed, and therefore that this monitoring system is necessary.

There have been some blockchain-based implementations for managing medical records. Ariel C Ekblaw designed MedRec—a decentralized record management system for EHRs—and implemented the pilot system in the Beth Israel Deaconess Medical Center [24]. Zhang et al [9] designed the architecture of DApp, named FHIRChain, based on 5 key requirements provided by the ONC interoperability roadmap and demonstrated the prototype. Roehrs et al [11,12] designed a distributed architecture model to integrate PHRs, which was called OmniPHR, and showed the performance of the prototypes. However, most of these models focus on data storage and sharing, whereas our study focused on monitoring changes in medical documents themselves.

There are some concerns about creating blockchain-based EHR systems because sensitive data—such as medical records—are protected by the General Data Protection Regulation legislation [25], which ensures that full control of data are given to data owners. Four rule-of-thumb principles that entrepreneurs and innovators can consider when designing blockchain-based apps have been proposed. Among these principles, the second states that personal data should be avoided on blockchain using data obfuscation, encryption, and aggregation techniques [26]. To comply with this principle, we used the hash value of the document using SHA-256—which is considered to be one of the most popular hashing algorithms in the world—to make it difficult to reverse the original.

Some blockchain systems ensure privacy and confidentiality using zero knowledge proofs (ZKPs) such as Zerocash [27]. ZKPs allow data to be verified without revealing the data. Although the app does not have access to the exact medical document contents, it can show whether the medical document has been changed or not. Our system therefore satisfies the concept of ZKPs because the app only identifies whether the item was changed or not, without knowledge of the item’s content.

Medical records may be often falsified to hide medical accidents [28] or to claim insurance by fraud [29]. In Korea, the Medical Service Act states that “Where any medical personnel or the founder of a medical institution makes an addition or revision to electronic medical records, he/she shall separately keep the access logs thereof, as prescribed by Ordinance of the Ministry of Health and Welfare” [30]. This act was implemented in September 2018 and the situation is likely to be similar in other countries. Most of the tertiary hospitals in Korea have well-integrated EHR systems that can manage changes in medical documents. However, primary care clinics use vendor-dependent EHR systems, which are not equipped to track changes in documents. Therefore, we chose a primary care clinic to trace changes in records.

### Limitations

This study has several limitations that should be noted. All logs in the system indicate that there were no changes to medical documents during the study period owing to the short duration and small sample size. In fact, there were not many documents in primary clinics to work with compared with those available at tertiary hospitals; the fewer the documents in primary clinics, the fewer the changes in documents. Nevertheless, this study demonstrated that the app functions as designed when using simulation data. Additionally, the selection of the hospitals was biased toward pediatric clinics. Finally, the SUS scores in this study were low. Because there were no changes in medical documents, the participants barely had a chance to realize the value of the app.

### Conclusions

This study introduced an app that notifies patients of changes in medical records using blockchain technology. Blockchain helps the app to inform patients of changes in their documents without uploading the medical record itself to the network. Therefore, blockchain can help ensure the transparency of medical records and advance patient empowerment.

## Appendix

Multimedia Appendix 1 FHIR Profiles of EHR Data.

Multimedia Appendix 2 Modified System Usability Scale (SUS).

Multimedia Appendix 3 Transaction metadata with simulation data.

Multimedia Appendix 4 Screenshots of medical document changes app tested with simulation data.

## Abbreviations

EHR: electronic health record
FHIR: Fast Healthcare Interoperability Resources
ONC: Office of the National Coordinator for Health Information Technology
PHR: personal health record
SUS: System Usability Scale
ZKP: zero knowledge proof
