# Supplementary figures and images for: Developing a Mobile App for Monitoring Medical Record Changes Using Blockchain: Development and Usability Study
Source: J Med Internet Res. 2020 Aug 14;22(8):e19657. doi: 10.2196/19657 (PMC7455865; doi:10.2196/19657)

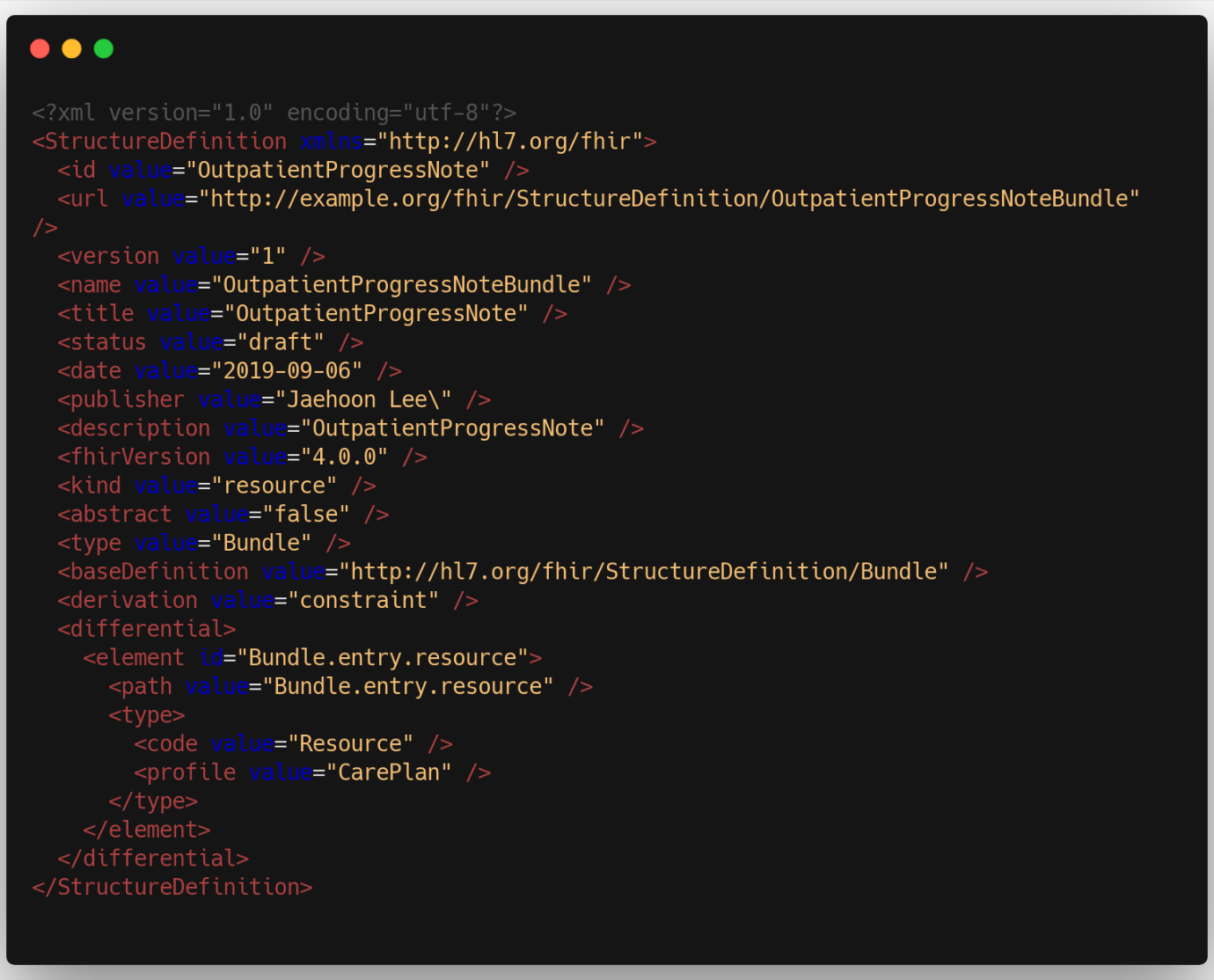

Supplement: Multimedia Appendix 1 [file jmir_v22i8e19657_app1.png]
