# Supplementary material for: Developing a Mobile App for Monitoring Medical Record Changes Using Blockchain: Development and Usability Study
Source: J Med Internet Res. 2020 Aug 14;22(8):e19657. doi: 10.2196/19657 (PMC7455865; doi:10.2196/19657)
Supplement: Multimedia Appendix 2 [file jmir_v22i8e19657_app2.docx]

**Multimedia Appendix 2**. Modified System Usability Scale (SUS)^a^

| (1) I think that I would like to use this product frequently. |
| --- |
| (2) I found the product unnecessarily complex. |
| (3) I thought the product was easy to use. |
| (4) I think that I would need the support of a technical person to be able to use this product. |
| (5) I found that various functions in this product were well integrated. |
| (6) I thought there was too much inconsistency in this product. |
| (7) I would imagine that most people would learn to use this product very quickly. |
| (8) I found the system very awkward to use. |
| (9) I felt very confident using the product. |
| (10) I needed to learn a lot of things before I could get going with the product. |

^a^The SUS showed the domains as five scales numbered from 1 (strongly disagree) to 5 (strongly agree). To obtain a score, the following formulas are used:

1. Positively worded domains = (score – 1).
2. Negatively worded domains = (score – 5).
3. After summing the ten domains, multiply by 2.5 = total SUS.
