# Supplementary material for: Developing a Mobile App for Monitoring Medical Record Changes Using Blockchain: Development and Usability Study
Source: J Med Internet Res. 2020 Aug 14;22(8):e19657. doi: 10.2196/19657 (PMC7455865; doi:10.2196/19657)
Supplement: Multimedia Appendix 3 [file jmir_v22i8e19657_app3.docx]

**Multimedia Appendix 3**. Transaction metadata with simulation data.

| **Simulation data** | **Medical record changes** | **Transaction Metadata_The original** | **Transaction Metadata_The changes** |
| --- | --- | --- | --- |
| Simulation 1 | 0 / 0 / 0 | 47D2A7D8E51B290065295EE225C62D86DC6FACD2760625C28C28CCD029E8BCBA |  |
| Simulation 2 | 0 / 0 / 3 | 79A68C1860C6B52C9D0C2F9A74808E0C2C816BAF2CC3378FE84DE2138A07BED0 | DD4D49D5415D08C86438A2CD51397C6C889DF35132F30E439D3B455228CE849B |
| Simulation 3 | 0 / 3 / 0 | 6CA2180BF78F1F2D2C012CC6C52BD34F8C6162AB124E57867686BEB936DCA9C5 | 28DC0DF8E17E2CD4FBDDB8351586D9A7ACAE4E7D6003B31F5D744E71F1FDE41D |
| Simulation 4 | 3 / 0 / 0 | 445BD02488497B7BC28CE0982CA361E1048ABDA6F562E13397DD349FCC7EA37F | 6D26FFC314F8B2B1631D4250A8C45BFCD2649FD5ED3A18600B638CA53BD22291 |
| Simulation 5 | 5 / 3 / 2 | 05C9FFE6AE372D90DEF5F46ADAE3C5ACFF0EB9C46B77D99702BC8031E4B990F0 | BA655E1CAFF2446031616D23C4D0D441680C900082518F423D58436B61BC56F1 |
