# Supplementary material for: Developing a Mobile App for Monitoring Medical Record Changes Using Blockchain: Development and Usability Study
Source: J Med Internet Res. 2020 Aug 14;22(8):e19657. doi: 10.2196/19657 (PMC7455865; doi:10.2196/19657)
Supplement: Multimedia Appendix 4 [file jmir_v22i8e19657_app4.docx]

**Multimedia appendix 4.** Screenshots of medical document changes app tested with simulation data.

| Simulation data | Medical record changes(risk1/risk2/risk3) | Screenshot |
| --- | --- | --- |
| Simulation 1 | 0 / 0 / 0 | 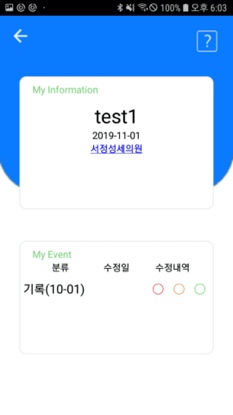 |
| Simulation 2 | 0 / 0 / 3 | 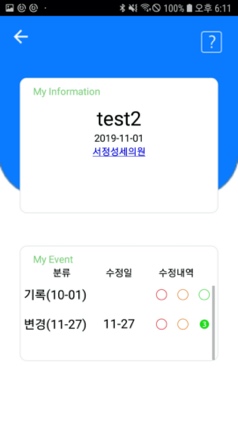 |
| Simulation 3 | 0 / 3 / 0 | 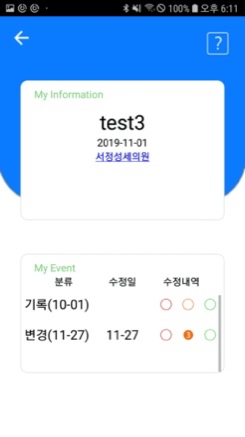 |
| Simulation 4 | 3 / 0 / 0 | 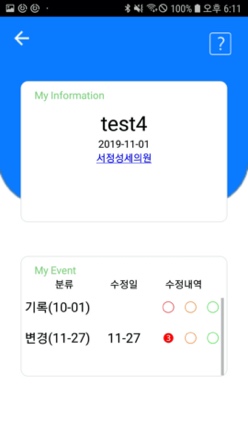 |
| Simulation 5 | 5 / 3 / 2 | 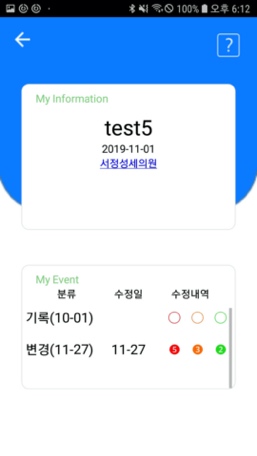 |
